# Angiomyolipomatous Lesions of the Nasal Cavity (Sinonasal Angioleiomyoma with Adipocytic Differentiation): A Multi-Institutional Immunohistochemical and Molecular Study

**DOI:** 10.21203/rs.3.rs-4843357/v1

**Published:** 2024-09-08

**Authors:** Victoria M. Jones, Lester D. R. Thompson, Jason R. Pettus, Donald C. Green, Joel A. Lefferts, Parth S. Shah, Gregory J. Tsongalis, Dipti P. Sajed, Julie M. Guilmette, James S. Lewis, Adam S. Fisch, Laura J. Tafe, Darcy A. Kerr

**Affiliations:** Dartmouth Hitchcock Medical Center; Head and Neck Pathology Consultations; Dartmouth Hitchcock Medical Center; Dartmouth Hitchcock Medical Center; Dartmouth Hitchcock Medical Center; Dartmouth Hitchcock Medical Center; Dartmouth Hitchcock Medical Center; University of California, Los Angeles; Hôpital Charles-Lemoyne, University of Sherbrooke; Vanderbilt University Medical Center; Massachusetts General Hospital, Harvard Medical School; Dartmouth Hitchcock Medical Center; Dartmouth Hitchcock Medical Center

**Keywords:** Nasal cavity, angioleiomyoma, angiomyolipoma, hamartoma, PEComa

## Abstract

**Purpose:**

Mesenchymal neoplasms composed of vascular, smooth muscle, and adipocytic components are uncommon in the nasal cavity. While angioleiomyoma (AL) is a smooth muscle tumor in the Head & Neck WHO classification, it is considered of pericytic origin in the Skin as well as Soft Tissue and Bone classifications. For nasal AL with an adipocytic component, the terms AL with adipocytic differentiation and angiomyolipoma (AML) have been applied, among others. AML is a type of perivascular epithelioid cell tumor (PEComa), most often arising in the kidney, sometimes associated with the tuberous sclerosis complex (TSC). It is uncertain whether nasal cavity AML and AL are best considered hamartomas or neoplasms, as their genetics are largely unexplored.

**Methods:**

We performed a multi-institutional retrospective study of nasal cavity mesenchymal lesions. Patient demographics, clinical histories, and histologic and immunohistochemical findings were collected. DNA and RNA were extracted from formalin-fixed, paraffin-embedded tissue and analyzed by SNP-based chromosomal microarray, targeted RNA fusion sequencing, and whole-exome sequencing.

**Results:**

Fifteen lesions (3 to 42 mm) were identified predominantly in male (87%) patients with a median age of 60. Patients typically presented with obstructive symptoms, and none had a history of TSC. One AL was a recurrence from six years prior; 11 cases showed no recurrence (median 4.7 years, range: 0.88–12.4). Morphologically, 11 AMLs contained 30–80% smooth muscle, 10–25% vasculature, and 2–60% adipose tissue, while four ALs contained 70–80% smooth muscle and 20–30% vasculature. Other histologic observations included surface ulceration, vascular thrombosis, chronic inflammation, and myxoid change; no well-developed epithelioid cell morphology was identified. Immunohistochemically, all cases were positive for smooth muscle markers (actin and/or desmin) and negative for melanocytic markers. Molecular analysis revealed loss of 3p and 11q in a single AML. No other known pathogenic copy number or molecular alterations were seen, including in *TSC1/2*, *TFE3*, or *NOTCH2*.

**Conclusion:**

Nasal cavity AML lacks morphologic, immunophenotypic, and genetic features of PEComa family AMLs. The significant histologic overlap between nasal AML and AL without distinguishing molecular features in either entity suggests “sinonasal angioleiomyoma with adipocytic differentiation” may be the most appropriate terminology for hybrid vascular and smooth muscle lesions containing adipocytic components.

## INTRODUCTION

Mesenchymal neoplasms composed of various proportions of vasculature and smooth muscle - with or without adipocytes - are exceedingly uncommon in the nasal cavity. Depending on the composition, variable terminologies are applied including nasal cavity angioleiomyoma (AL) [1–2], AL with adipocytic differentiation [3–5], and angiomyolipoma (AML) [6–12]. While AL is considered a pericytic tumor in the WHO Skin and Soft Tissue and Bone tumor classification systems [13–14] and a smooth muscle tumor in the WHO Head and Neck tumor classification [15], AML is not recognized as an entity in these classifications. However, in the WHO Urinary and Male Genital tumors classification [16], AML is listed as a subtype of extra-renal pericytic tumors while in the Digestive System tumors classification it is considered a tumor of uncertain differentiation [17]. Overall, it is unclear whether these mesenchymal growths in the nasal cavity represent neoplasms or hamartomas (i.e., an abnormal/disorganized proliferation of cells normally found in the anatomic region).

AML is better known as a member of the perivascular epithelioid cell tumor (PEComa) family, most often arising in the kidney or liver. These tumors demonstrate a triphasic morphologic pattern with variable amounts of smooth muscle, thick-walled blood vessels, and mature adipose tissue. In addition to epithelioid PEComa variants, other lesions within this spectrum include soft tissue PEComa, pulmonary PEComa (clear cell ‘sugar’ tumor of the lung), and lymphangioleiomyomatosis [18]. These various types of PEComas share morphologic features, namely perivascular epithelioid cell differentiation, and immunoreactivity for melanocytic markers (HMB45/Melan A), supported by ultrastructural evidence of premelanosome-like granules [19], as well as smooth muscle markers (actin/desmin). AML may arise sporadically or through germline mutations in the tuberous sclerosis complex 1 (*TSC1*) or 2 (*TSC2*) genes on chromosomes 9q and 16p, respectively [20–21]. In sporadic cases, biallelic inactivation of *TSC2* or *TSC1* occurs through variable genetic events (most commonly point mutations), whereas in the syndromic setting the second allele is commonly inactivated through loss-of-heterozygosity [22].

In contrast, AML of the nasal cavity is exceptionally rare [11] and generally considered a non-PEComatous entity. In a recent case report with literature review, Wang, et al. characterized nasal cavity AML as a tumor containing spindled smooth muscle cells lacking epithelioid morphology, mature adipose tissue, and thick-walled vessels without a melanocytic immunophenotype [9]. The genetic underpinnings of these nasal mesenchymal tumors are hitherto unexplored in the literature. We aim to use molecular analysis to determine the clonal/neoplastic status of nasal angiomyolipomatous lesions, to better understand whether AL and AML are distinct entities, and to identify any relationship between nasal AML and PEComa family tumors associated with tuberous sclerosis complex (TSC).

## MATERIALS AND METHODS

### Multi-institutional clinicopathologic study

After approval from the respective Institutional Review Boards of each of the six participating institutions, the anatomic pathology archives were searched from January 2011 to October 2022 for sinonasal mucosal lesions coded with terms such as: angiomyolipoma, hamartoma, angiomyoma, or angioleiomyoma. Patient demographics, clinical histories, and radiographic findings were collected from available electronic medical records. The histopathologic features and immunohistochemical results were recorded based on review of the available pathology slides and reports. Ultimately, diagnostic confirmation or re-classification based on histologic components was performed and agreed upon by two study authors (VMJ and DAK).

### Histopathologic evaluation

Available hematoxylin and eosin-stained and immunohistochemical slides were retrieved and reviewed by the study authors. The following features were recorded: estimated percentages of smooth muscle, vasculature, and adipose components; surface ulceration; growth pattern; margin of the lesion; the presence or absence of epithelioid cells, mitotic activity, and necrosis; and any other notable histologic features. Given the lack of a consensus definition within the WHO Classifications or the broader literature, we classified tumors based on the mesenchymal components present; those composed of smooth muscle and vasculature were classified as angioleiomyoma (AL), and those with the additional presence of any amount of adipose tissue as were classified as angiomyolipoma (AML). A representative formalin-fixed, paraffin-embedded (FFPE) tissue block for each patient was identified and retrieved for additional immunohistochemical and molecular testing.

### Immunohistochemical analysis

Immunohistochemistry was performed on 4 μm FFPE sections using commercially available antibodies. Standard autostaining protocols were followed (DAKO EnVision^™^+ System (Agilent Technologies, Santa Clara, CA) and Leica BOND-III (Leica Biosystems, Buffalo Grove, IL)). Three cases were performed at the originating institution as a part of clinical care. In the remaining cases, immunochemical analysis was performed at Dartmouth Hitchcock Medical Center. Antibodies used included α-Smooth Muscle Actin (SMA) (DAKO, mouse monoclonal, 1:150 dilution) and HMB45 (DAKO, mouse monoclonal, 1:125 dilution). Appropriate negative and positive controls were included (smooth muscle for SMA and melanoma for HMB45).

### Molecular methods

DNA and RNA were extracted from FFPE tissue blocks following the Qiagen AllPrep extraction kit protocol (Qiagen, Germantown, MD). To assess for monosomy, copy number variation, and loss of heterozygosity, the OncoScan^™^ CNV Assay (Thermofisher, Waltham, MA), a single nucleotide polymorphism (SNP)-based chromosomal microarray, was utilized. Additionally, targeted next-generation sequencing of RNA fusions using the TruSight Tumor 170 assay (Illumina, San Diego, CA) and DNA whole exome sequencing using the SureSelect Human All Exon V8 panel and Magnis NGS Prep System (Agilent Technologies, Santa Clara, CA) were performed. Single indexed libraries were sequenced on the Illumina Nova-Seq 6000. Sequencing data and variant analysis was performed using AUGMET, a validated in-house comprehensive bioinformatics platform for genomic data [23]. DNA exome sequencing data analysis was filtered using a set of 55 cancer-related genes.

## RESULTS

### Clinical and radiographic characteristics

**Figure 1:**
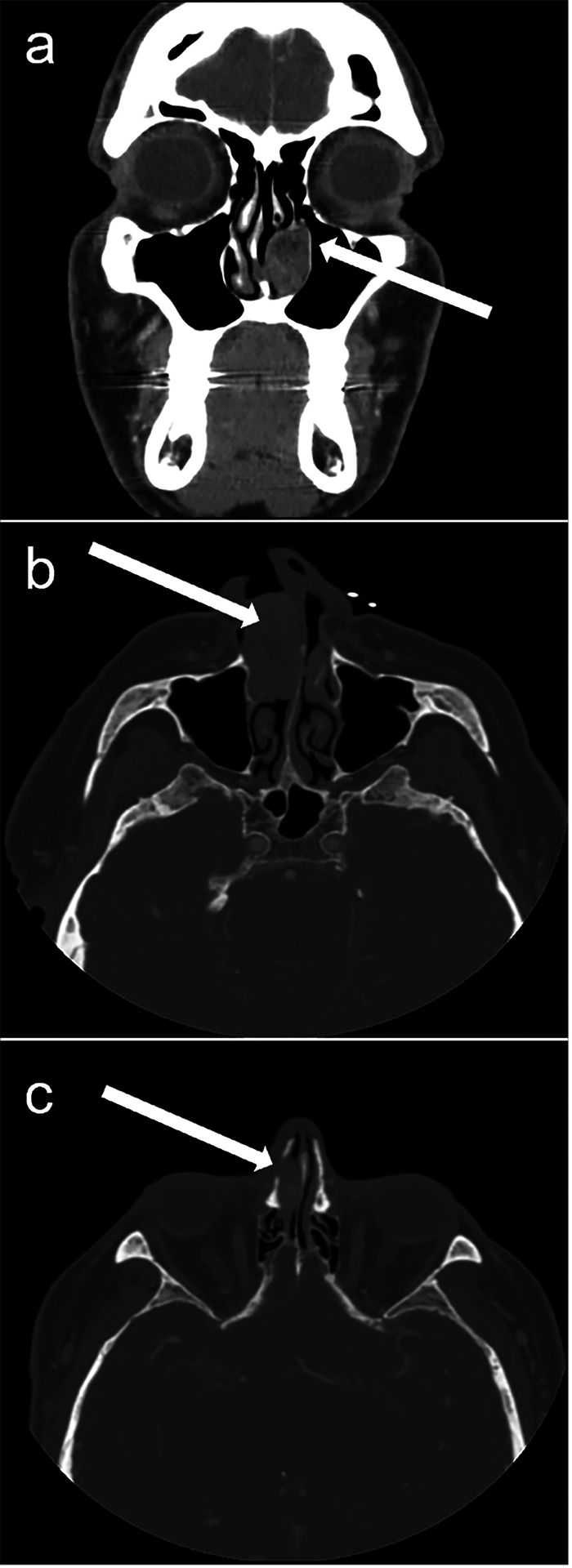
Radiographic features of nasal cavity angiomyolipomatous lesions. Computed tomography images portraying, by white arrows, a left nasal lacrimal duct mass with osseous remodeling (patient #2) (a) and a right nasal mass (b) with new sites of osseous erosion (c) (patient #1)

Fifteen patients with nasal cavity lesions were identified from 2011 to 2022 using the established search criteria. The demographics, clinical presentation, tumor location and size, radiographic characteristics, and surgical pathology diagnoses are shown in Table 1. In brief, 13 male and two female patients were included with a median age of 60 years. The lesions ranged in size from 3 to 42 mm and originated from the inferior turbinate (n = 3), nasal cavity wall (n = 2), nasal floor (n = 2), septum (n = 2), inferior meatus (n = 1), and sinonasal tract not-otherwise specified (NOS) (n = 1). Fourteen patients had no history of TSC (history was unknown for one). Patients presented with obstructive symptoms (n = 10), as well as epistaxis (n = 6), congestion (n = 4), nasal polyps (n = 2), yellow discharge (n = 1), increased postnasal drip and sinus pressure (n = 1), headaches (n = 1), and nasal valve incompetence (n = 1). Radiographic imaging revealed mucosal thickening (n = 3), bony erosion (n = 2), osseous remodeling (n = 1) (Fig. 1). Following surgical excision or resection, the lesions at each participating institution were initially diagnosed under variable diagnostic terminologies including: “benign soft tissue neoplasm, consistent with AML,” “sinonasal AL with adipocytic differentiation,” “benign myoid tumor suggesting an AL,” “AML of nasal cavity,” and “AL (vascular leiomyoma).” In total, after strictly defining ALs as being composed of smooth muscle and vasculature only, and those with the additional presence of any amount of adipose tissue as AML, 11 sinonasal AML (9:2 M:F, average 2.2 cm) and four AL (4:0 M:F, average 2.2 cm) were examined. Of note, five cases initially submitted as AL were re-classified as AML based on the presence of adipose tissue. One AL (patient #12) was a recurrence from six years prior; 11total cases with subsequent follow-up showed no recurrence (median 4.7 years, range: 0.88–12.4). Specifically, eight AML showed no recurrence (median 4.3 years, range: 0.9–9.2), and three AL showed no recurrence (median 8.9 years, range: 1.8–12.4).

### Histopathologic and immunohistochemical features

**Figure 2:**
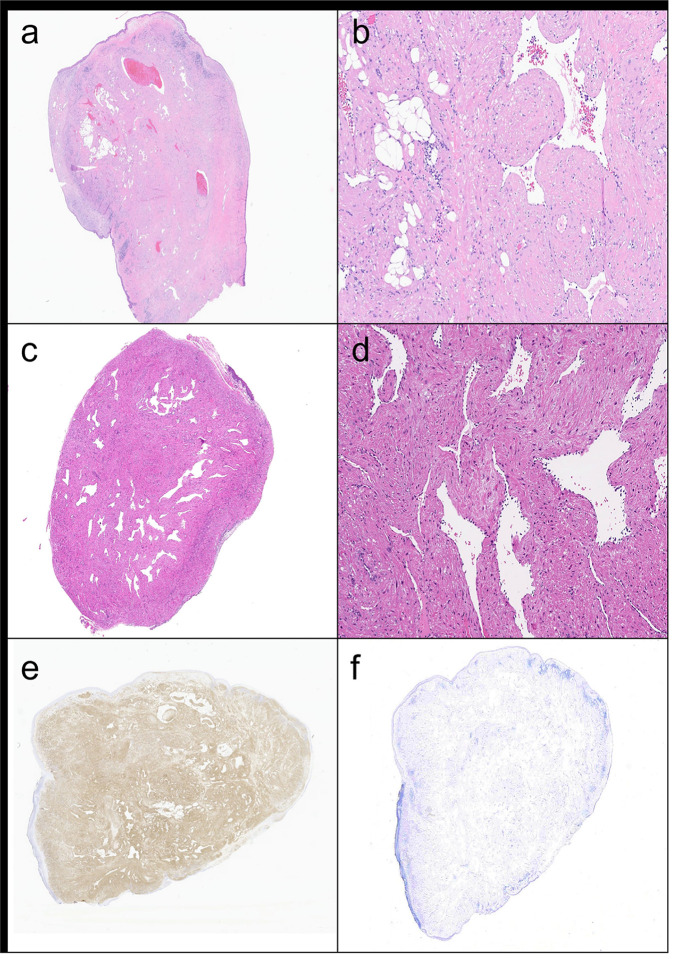
Histologic and immunophenotypic features of nasal cavity angiomyolipomatous lesions. Low and high-power magnifications of hematoxylin and eosin-stained sections of angiomyolipoma (a-b, patient #1) and angioleiomyoma (c-d, patient #13). By immunohistochemistry, all cases were positive for smooth muscle markers (e: actin, patient #1) and negative for melanocytic markers (f: HMB45, patient #1)

**Figure 3:**
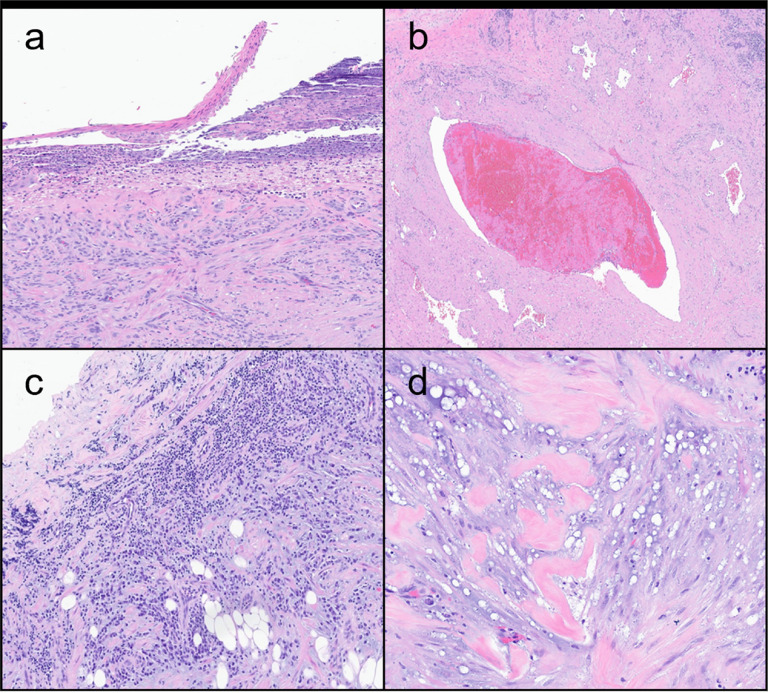
Additional histologic features of nasal cavity angiomyolipomatous lesions. Variable histologic findings include necrosis associated with surface ulceration (a, patient #1), vascular thrombosis (b, patient #1), chronic inflammation (c, patient #2), and myxoid change with keloidal collagen (d, patient #14)

Eleven AMLs (Fig. 2A–B) contained 10–25% vasculature, 30–80% smooth muscle, and 2–60% adipose tissue, while four ALs (Fig. 2C–D) contained 20–30% vasculature and 70–80% smooth muscle (Table 2). Surface ulceration (n = 5) and necrosis (n = 3) and were present in AMLs only. In three cases, necrosis was associated with surface ulceration (Fig. 3A). Tumors generally demonstrated a circumscribed border (n = 8), with infiltrative growth only seen in two AMLs. The surgical margins of both AML and AL lesions were typically positive (n = 10). In five cases, growth pattern and/or margin status could not be confidently assessed due to cautery, fragmentation, or irregular distribution of mesenchymal components. Well-developed epithelioid cell morphology or mitotic activity was not identified in any case. Common histopathologic features across all lesions included vascular thrombosis, chronic inflammation, myxoid stromal change, and keloidal-type collagen (Fig. 3B–D), as well as vascular changes (slit-like or staghorn-type vessels). Rarely, extramedullary hematopoiesis, squamous metaplasia near surface ulceration, or enlarged senescent-type cytologic atypia was observed. All cases were positive for muscle markers (SMA [n = 13], Fig.2E, and/or desmin), negative for melanocytic markers (HMB45 [n = 14], [Figure 2F], SOX10 [n = 5], Melan A [n = 3], MART1 [n = 1], and MITF [n = 1]), and negative for keratin AE1/AE3 (n = 2). Additionally, S100 was utilized as both an adipocytic marker (positive, n = 2) and melanocytic marker (negative, n = 3).

### Molecular findings

**Figure 4:**
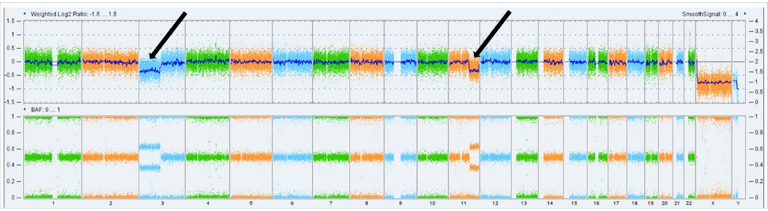
Whole-genome image view of the single nasal angiomyolipoma case (patient #5) that demonstrated clear copy number variations by SNP-based chromosomal microarray (Top plot: Log2 Ratio; Bottom plot: B-allele frequency [BAF]). Two losses/deletions are present, 3p and 11q (arrows)

Extracted DNA and RNA was sufficient for SNP array analysis in 14 cases (one case failed) and NGS in all 15 cases (one case was RNA-only as DNA extraction failed). Molecular analysis revealed loss of 3p and 11q in a single AML (patient #5, Table 2, Fig. 4). Additional low-level (subclonal) loss on chromosome 15 in another AML may have been possible but was below our limit of detection (~ 20% tumor cells). A few variants of unknown significance were identified (Table 2). No other CNV, LOH regions, or classic molecular alterations (including in *TSC1/2*, *TFE3*, or *NOTCH2*) were seen. Tumor mutational burden was low in all cases.

## DISCUSSION

Angiomyolipomatous lesions in the nasal cavity such AML and AL, which are composed of smooth muscle and vasculature with and without adipocytes, have been sparsely characterized in the literature. AL is described as a subtype of leiomyoma within the WHO Head and Neck smooth muscle tumor category. It is exceedingly rare in the nasal cavity. It is unknown whether nasal AL harbors similar or divergent molecular alterations to AML. Alterations associated with the PEComatous renal and hepatic AML include biallelic loss of the tumor suppressor genes, *TSC1* or *TSC2*, or, alternatively, *TFE3* gene fusions [24]. Genes or pathways reported as implicated in AL include monosomy of chromosome 13; loss of 6p, 13q, 21q, and 22q; recurrent gain at Xq; and rarely, *NOTCH2* gene arrangement [25–31]. The *MED12* and *HMGA2* rearrangements of genital and retroperitoneal leiomyomas have not been reported in sinonasal leiomyomas [32–33]. Due to the rarity of these nasal cavity lesions, the literature and experience are largely limited to case reports. To our knowledge, our research is first to evaluate the genetic landscape of angiomyolipomatous lesions of the nasal cavity through SNP array, whole exome sequencing, and RNA sequencing.

In our case series of 15 nasal cavity angiomyolipomatous lesions (11 AML and four AL, mean size 22 mm), there was a striking 87% male patient predominance with no clinical history of TSC. Radiographic studies revealed variable features of bony erosion, osseous remodeling, and mucosal thickening. Most tumors demonstrated a circumscribed growth pattern. Surface ulceration and associated surface necrosis, vascular thrombosis, chronic inflammation, myxoid change, and vascular changes were seen in a subset of cases. Well-developed epithelioid cell morphology or melanocytic immunophenotypic signatures were not identified. Despite typically positive surgical margins, we observed no case recurrences during the study follow-up period, and only one case (patient #12) clinically represented recurrent disease. In summary, we confirmed that AML in the nasal cavity, compared to the more common kidney or liver locations, lacks epithelioid morphology, melanocytic immunoexpression, and association with TSC; it is indeed thereby best considered as a non-PEComatous entity [18–19]. We also suggest that nasal “AML” and “AL” are likely the same entity, given the significant histologic overlap and immunophenotypic profile and no clinically relevant, distinguishing genetic features.

Molecular analysis revealed loss of 3p and 11q in a single AML (patient #5, Fig. 4). Interestingly, this was the eldest patient at 75 years (study mean age, 60 years) and was one of only two patients that exhibited bony erosion on radiographic imaging, though the size of his AML and clinical history are unknown. The clinical significance of the 3p and 11q loss is unclear, as findings have not otherwise been reported in angiomyolipomatous lesions. However, in more than 90% of sporadic clear cell renal cell carcinomas, loss of chromosome 3p, which harbors tumor suppressors *VHL* on 3p25 and *PBRM1*, *BAP1*, and *SETD2* on 3p21, is an established occurrence [34]. Deletion of chromosome 11q is implicated in neuroblastoma, conferring poorer prognosis in high-risk patients [35]. Deletion of 3p is also shown to be nonrandomly associated with deletion of 11q in neuroblastoma [36]. Overall, no other known pathogenic CNV, LOH regions, or classic molecular alterations were seen, including in *TSC1/2*, *TFE3*, and *NOTCH2*. One variant of uncertain significance (VUS) involving *TSC2* was identified (patient #15). This *TSC2* variant is a splice site donor (with one report in ClinVar [Variation ID: 2683333]). Similar splice variants in this region are considered benign. Several other VUS were also identified (Table 2), two of which were seen in genes associated with the androgen receptor (*NCOR1* and *MDC1*) [37–38]. While these variants are insufficiently characterized in existing literature and genomic databases to ascertain their potential biologic significance, in the context of the predominant male predilection noted clinically, the possibility of a hormonal response component in the pathogenesis of these lesions is raised. This response may be possibly analogous to sinonasal tract angiofibroma, a lesion that exclusively affects male patients and demonstrates a hormone dependent growth pattern correlated with puberty onset and strong androgen receptor expression [39–41].

Entities such as AML and AL pose a diagnostic challenge with regard to developing consistent nomenclature and defining them as either neoplastic or hamartomatous. Derived from the Greek root *hamartia* (“to miss the mark”), hamartomas consist of an abnormal proliferation of cells normally found in an anatomic region but failing to form the structures expected for the region [42]. Some authors also indicate that hamartomas should show evidence of being present near the time of birth [43]. Hamartomas may also show clonality, which blurs the distinction from true neoplasms. The WHO Head and Neck tumor categories of nasal hamartomas currently includes only respiratory epithelial adenomatoid hamartoma (REAH), seromucinous hamartoma (SH), and nasal chondromesenchymal hamartoma (NCMH) [44]. The unusually high fractional allelic loss of 31% for REAH suggests it may be a benign neoplasm [45], though recent evidence show its lack of *KRAS*, *BRAF*, or *EGFR* mutations [46]. There have been documented *EGFR::ZNF267* gene fusions [45] and increased mutation rates in heteroplasmy [47] in SH, suggesting it as a benign neoplasm. Thirdly, NCMH has been shown to exhibit somatic *DICER1* missense mutations [48] as well as a t(12;17)(q24.1;q21) translocation [49], classifying it as a benign neoplasm of nasopharynx, though the terminology of hamartoma is retained. Therefore, each of the three WHO-classified hamartomas of the nasal cavity exhibit unique genetic aberrations. However, in contrast to neoplasms, hamartomas typically have self-limited growth and do not recur [50]. For example, in a retrospective study of sinonasal REAH, 49 cases were endoscopically resected without recurrence in a mean follow-up period of 27.2 months [51]. In this delicate anatomic location, where even small lesions present with symptoms (nasal obstruction), surgical excision may be favored clinically over observation (unlike renal or liver AMLs). Resections of hamartomas appear to be curative [51]. Similarly, in the largest series of sinonasal angioleiomyomas, no recurrences occurred after local excision [5].

The classification of nasal tumors with features of non-PEComatous AML has been inconsistent and controversial in the literature. Diagnostic proposals have included: “angiomyolipomatous hamartoma”, wherein authors favored these termed lesions as non-PEComatous and likely non-neoplastic, though molecular testing was not performed [9]; “mucocutaneous AML” [12], “nasal AML”, and “sinonasal AL with adipocytic differentiation” [3]. In a series of 16 sinonasal ALs, authors noted that 25% demonstrated mature adipocytes and in reviewing the literature noted adipocytes in a higher proportion (35%) of sinonasal ALs compared to the cutaneous counterparts (2.8%) [5]. Furthermore, AL with adipocytic differentiation tended to affect males more (63%) compared to AL without adipocytic differentiation (46%) [5]. In our multi-institutional study with multiple participating pathologists, we noted variability in the diagnostic nomenclature used in practice for these lesions. Five cases, initially submitted as “sinonasal AL with adipocytic differentiation” and “AL (vascular leiomyoma)”, were re-classified to AML once defining lesions with any amount of adipose tissue as AML for the purposes of this study. However, we do note the controversy of simply labeling these nasal cavity lesions as “AML”, as cautioned by Tosios, et al. [52]: AML is a well-studied and defined entity to clinicians, conferring specific diagnostic value to clinicians and patients that may otherwise mislead.

In conclusion, angiomyolipomatous lesions in the nasal cavity warrant a universally adopted nomenclature. Genetic analysis shows that sinonasal angiomyolipomatous lesions distinct from neoplastic, PEComatous angiomyolipomatous lesions of the kidney, lacking alterations in the known PEComa-related genes. Therefore, while these benign nasal cavity lesions could reasonably descriptively be termed as “angiomyolipomatous hamartomas” [9], this nomenclature may be misleading because of its tendency to invoke associations with PEComatous AML. Most of these lesions affect adults without evidence of origin near the time of birth, calling into question characterization as hamartomatous. Thus, it may be most prudent to endorse the nomenclature of “sinonasal angioleiomyoma with adipocytic differentiation.”

## Figures and Tables

**Table 1:** Clinical findings of 15 sinonasal tract angiomyolipomatous lesions.

Patient	Age (yr)/Sex	Site	Size (mm)	Obstructive symptoms?	Radiographic characteristics	Surgical procedure	Surgical diagnosis	Study diagnosis (re)classfication	Length of follow-up (yr); recurrence?
1	70/M	Lateral wall	37	Yes	Bony erosion	En bloc resection	“Benign soft tissue neoplasm, consistent with angiomyolipoma”	AML	0; N/A
2	41/M	Inferior meatus	24	Yes	Osseous remodeling	Inferior turbinate excision	“Benign soft tissue neoplasm, consistent with angiomyolipoma”	AML	0; N/A
3	47/M	Inferior turbinate	7	Yes	N/A	Inferior turbinate resection	“Angiomyolipoma of nasal cavity”	AML	0.88; No
4	57/M	Floor	7	No	N/A	Endoscopic resection	“Angiomyolipoma of nasal cavity”	AML	4.79; No
5	75/M	Floor	N/A	N/A	Enhancing solid mass with bony erosion	Endoscopic resection	“Sinonasal angioleiomyoma with adipocytic differentiation”	(AML)	0.03; N/A
6	41/M	Inferior to nasal duct opening	40	Yes	Lesion appears adherent to inferior turbinate; no bony erosion	En bloc resection	“Benign myoid tumor suggesting an angioleiomyoma”	AML	4.74; No
7	60/F	Lateral anterior wall	12	Yes	Mucosal thickening; no bony destruction	Excision	“Angiomyolipoma of nasal cavity”	AML	3.91; No
8	32/M	Right skull base, sinonasal tract	42	Yes	No bony destruction	Excision	“Angioleiomyoma (vascular leiomyoma)”	(AML)	2.26; No
9	35/M	Left nasal cavity	12	Yes	N/A	Excision	“Angioleiomyoma (vascular leiomyoma)”	(AML)	4.69; No
10	66/M	Inferior turbinate	25	Yes	N/A	Excision	“Angioleiomyoma (vascular leiomyoma)”	(AML)	9.21; No
11	64/F	Right nasal cavity	9	No	Mucosal thickening with right nasal cavity abnormality	Endoscopic resection	“Consistent with angioleiomyoma”	(AML)	1.12; No
12	64/M	Septum	3	No	N/A	Endoscopic resection	“Angioleiomyoma”	AL	N/A; Yes (from 2009)
13	55/M	Septum	40	Yes	Mucosal thickening	Excision and curettage	“Angioleiomyoma (vascular leiomyoma)”	AL	12.4; No
14	66/M	Inferior turbinate	28	Yes	N/A	Excision	“Angioleiomyoma (vascular leiomyoma)”	AL	8.89; No
15	61/M	Right anterior nasal cavity	15	No	Enhancing lesion at anterior face of inferior turbinate	Endoscopic resection	“Angioleiomyoma”	AL	1.79; No

AML: angiomyolipoma, AL: angioleiomyoma, N/A: not available, M: male, F: female; yr, years.

**Table 2:** Histopathologic and molecular features of study cohort lesions.

	% Composition			Histologic features (P, present; A, absent)					Molecular features	
Patient	Vascular	Smooth muscle	Adipose	Necrosis	Surface ulceration	Surgical margin	Growth pattern (I, infiltrative; C, circumscribed)	Other histologic features	Microarray	NGS (VUS)
1	15	75	10	P	P	Negative	C	Extramedullary hematopoiesis, vascular thrombosis	-	-
2	10	30	60	P	P	Positive	N/A	Chronic inflammation, extravasated RBCs, myxoid stromal change, keloidal-type collagen (nodular fasciitis-like)		*NCOR1* p.Arg1229Gln; *NCOR1* p.His2252Ty (43–47%)
3	20	75	5	A	P	Positive	C	N/A	Failed; insufficient DNA/RNA	*MAP3K4* p.Leu1531ArgfsTer1 (9.5%)
4	15	45	40	A	A	Positive	I	Chronic and focally acute inflammation	Possible 5.5 Mb deletion distal 15q (very low level, near LOD)	-
5	25	65	10	A	A	Positive	N/A	Thrombosis and hemosiderin deposition	3p loss (entire short arm); 11q loss (distal half)	-
6	25	70	5	P	P	N/A	N/A	Chronic inflammation, vascular thrombosis, extravasated RBCs and hemosiderin deposition, squamous metaplasia (at surface near ulcer)	-	-
7	15	70	15	A	A	Positive	C	Chronic inflammation, myxoid change	-	-
8	10	80	10	A	A	Positive	I	N/A (cauterized)	-	*MDC1* p.Gly207_Phe214del (32.5%)
9	18	80	2	A	P	Negative	C	Chronic inflammation, focal acute inflammation, focal thrombosis		
10	20	75	5	A	A	Positive	N/A	Fragmented. Irregular fat distribution.	-	-
11	20	60	20	A	A	Positive	C	Scattered enlarged senescent-type cells, chronic sinusitis	-	Failed DNA extractio (RNA only)
12	25	70	0	A	A	N/A	N/A	Squamous metaplastic	-	-

RBCs: red blood cells; N/A: not applicable due, but not limited, to cautery, fragmentation, or irregular distribution of mesenchymal components; NGS: next-generation sequencing; LOD: limit of detection; SNP: single nucleotide polymorphism; VUS: variants of unknown significance

## Data Availability

Data/materials are available upon reasonable request via the corresponding author.
